# Poor Cerebral Inflammatory Response in eIF2B Knock-In Mice: Implications for the Aetiology of Vanishing White Matter Disease

**DOI:** 10.1371/journal.pone.0046715

**Published:** 2012-10-04

**Authors:** Yuval Cabilly, Mali Barbi, Michal Geva, Liraz Marom, David Chetrit, Marcelo Ehrlich, Orna Elroy-Stein

**Affiliations:** 1 Department of Cell Research and Immunology, George S. Wise Faculty of Life Sciences, Tel Aviv University, Tel Aviv, Israel; 2 Sagol School of Neuroscience, Tel Aviv University, Tel Aviv, Israel; Baylor Research Institute, United States of America

## Abstract

**Background:**

Mutations in any of the five subunits of eukaryotic translation initiation factor 2B (eIF2B) can lead to an inherited chronic-progressive fatal brain disease of unknown aetiology termed leucoencephalopathy with vanishing white matter (VWM). VWM is one of the most prevalent childhood white matter disorders, which markedly deteriorates after inflammation or exposure to other stressors. eIF2B is a major housekeeping complex that governs the rate of global protein synthesis under normal and stress conditions. A previous study demonstrated that Eif2b5^R132H/R132H^ mice suffer delayed white matter development and fail to recover from cuprizone-induced demyelination, although eIF2B enzymatic activity in the mutant brain is reduced by merely 20%.

**Principal Findings:**

Poor astrogliosis was observed in Eif2b5^R132H/R132H^ mice brain in response to systemic stress induced by peripheral injections of lipopolysaccharide (LPS). Even with normal rates of protein synthesis under normal conditions, primary astrocytes and microglia isolated from mutant brains fail to adequately synthesise and secrete cytokines in response to LPS treatment despite proper induction of cytokine mRNAs.

**Conclusions:**

The mild reduction in eIF2B activity prevents the appropriate increase in translation rates upon exposure to the inflammatory stressor LPS. The data underscore the importance of fully-functional translation machinery for efficient cerebral inflammatory response upon insults. It highlights the magnitude of proficient translation rates in restoration of brain homeostasis via microglia-astrocyte crosstalk. This study is the first to suggest the involvement of microglia in the pathology of VWM disease. Importantly, it rationalises the deterioration of clinical symptoms upon exposure of VWM patients to physiological stressors and provides possible explanation for their high phenotypic variability.

## Introduction

A wide range of known mutations in each of the five subunits of eukaryotic translation initiation factor 2B (eIF2B1-5) can lead to a fatal autosomal-recessive neurodegenerative disorder that primarily affects the white matter of the central nervous system (CNS), termed vanishing white matter disease (VWM, OMIM #603896), childhood ataxia with CNS hypomyelination (CACH) or eIF2B-related leucodystrophy [1]–[4]. VWM disease, one of the most prevalent inherited childhood white matter disorders, shows a wide variability in disease onset and symptom severity, which depend on the patient’s specific eIF2B mutation, genetic background and exposure to environmental stressors. The classic form of the disease is associated with a progressive loss of cerebral white matter as detected by magnetic resonance imaging, which leads to neurological, motor and cognitive deficits. Episodes of severe clinical deterioration are often observed upon exposure to various stressors, such as febrile illness, head trauma and acute fright, resulting in death by adolescence [4]–[8]. VWM is thought to predominantly affect oligodendrocytes and astrocytes, while microglial cells were thus far not considered to play a part in the pathogenesis of the disease. The neuropathology is characterized by abnormal “foamy” oligodendrocytes and increased density of immature oligodendroglial cells. Astrocytes have maturation defects and atypically low astrogliosis with coarse, blunted processes and anomalous composition of intermediate filament network [9]–[13].

eIF2B is an essential regulator of protein synthesis that serves as the guanine nucleotide exchange factor (GEF) of eukaryotic translation initiation factor 2 (eIF2). In its GTP-bound form, eIF2 binds aminoacylated initiator methionyl-tRNA to generate eIF2-GTP-tRNA_i_^Met^ ternary complex, which then interacts with the small ribosomal subunit. Following the release of GDP-eIF2 in each round of translation initiation, eIF2 is recycled to its active GTP-bound form by eIF2B. The formation of ternary complexes and thus the rate of global protein synthesis directly depend on eIF2B activity [14], [15]. eIF2B is also responsible for attenuation of global protein synthesis under various forms of cellular stress. This is achieved via activation of one of four kinases that phosphorylate the α-subunit of eIF2, which then acts as a competitive inhibitor of eIF2B [16]–[19].

Astrocytes, the most abundant cell type in the central nervous system, have multiple support functions that help maintain brain homeostasis [20]. The process of astrocyte activation, termed astrogliosis, is elicited by a wide range of brain insults and is characterized by hypertrophy and increase in the number of cellular activities due to increased expression of multiple proteins such as intermediate filaments (including glial fibrillary acidic protein, GFAP), among others. Mild-to-moderate reactive astrogliosis is elicited by mild non-penetrating head trauma, viral or bacterial infections and exposure to circulating antigens or endotoxins e.g. lipopolysaccharide (LPS) due to peripheral infections. Reactive astrocytes exert pro- and anti-inflammatory activities by secreting a diverse repertoire of cytokines, chemokines and neurotropic factors that promote tissue repair and remyelination [21], [22]. Brain homeostasis also depends on the function of the resident macrophage-like microglial cells, which play an important role in triggering and modulating astrogliosis. In response to injury or infection, microglial cells respond more rapidly and intensively than astrocytes and secrete cytokines that bind to receptors on the cell surface of astrocytes. Pro-inflammatory cytokines secreted from activated microglial cells can affect the activation of astrocytes. In response to this stimuli, astrocytes produce and secrete cytokines that act on microglia, thus creating a paracrine and autocrine feedback loop whereby microglial- and astroglial-derived factors regulate each other [23].

In a previous study, a mutant mouse model for VWM disease was generated by introducing an R132H mutation into the mouse Eif2b5 gene locus. This mutation corresponds to the R136H mutation in the human gene, which is associated with the classic form of VWM disease when present in a homozygous state. In brain lysates of Eif2b5^R132H/R132H^ mice, the GEF activity of eIF2B is reduced by approximately 20%. Eif2b5^R132H/R132H^ mice exhibit delayed development of brain white matter, higher proportion of small-calibre nerve fibres, abnormal abundance of oligodendrocytes and astrocytes in younger animals, abnormal levels of major myelin proteins and early neurodegeneration. Moreover, the mutant mice fail to recover from cuprizone-induced demyelination, reflecting an increased sensitivity to brain insult and difficulty repairing damaged myelin [24].

In the current study, we used the Eif2b5^R132H/R132H^ mice to test our hypothesis that this mutation in eIF2B is associated with impaired activation of astrocytes and microglia. To this end, we used lipopolysaccharide (LPS), a pyrogenic agent, which also activates astrocytes and microglia cells in culture. The impaired astrogliosis was evident at the whole-animal level, where systemic LPS injection led to a lower induction of GFAP and IL-6 protein in the brains of Eif2b5^R132H/R132H^ mice compared to normal mice. Mutant primary astrocytes isolated from mice brains fail to undergo proper activation in response to LPS. Whereas the mild reduction in eIF2B activity does not decrease the rate of protein synthesis under normal conditions, it does prevent the normal increase in translation rates upon exposure to LPS. This anomaly is evident by the lower synthesis rate of multiple cytokines in response to LPS treatment, despite similar induction of transcription of the corresponding mRNAs. Moreover, mutant microglia suffer a similar impairment, implicating their involvement in the poor astrogliosis. These results are the first to suggest a role for microglia in VWM pathology, and the first to relate poor performance of the neuroinflammatory system to the aetiology of the disease.

## Results

### Astrogliosis is Impaired in Eif2b5^R132H/R132H^ Mice

**Figure 1:**
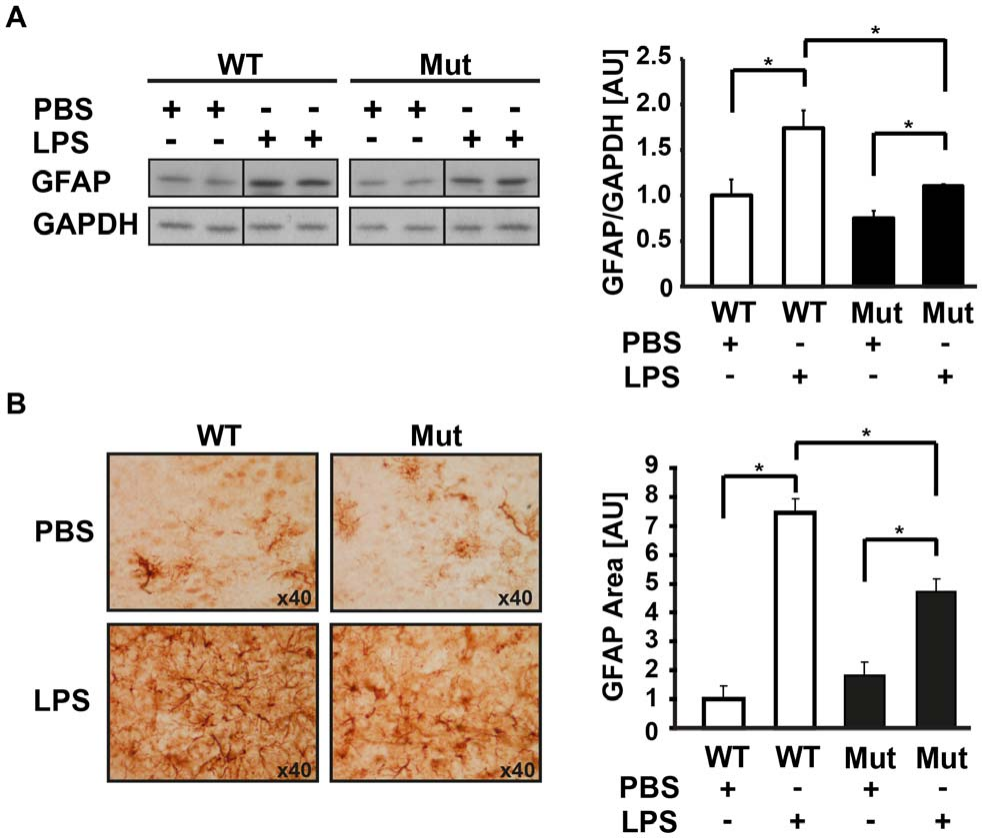
eIF2B5^R132H/R132H^ mice exhibit impaired astrogliosis. Intraperitoneal injections of 5 mg/kg LPS or PBS were administered to 7 Eif2b5^R132H/R132H^ (Mut) and 7 wild-type (WT) mice at P14 and again at P15. At P21 mice were sacrificed and brains were removed. (A) Representative immunoblot analysis of whole brain using anti-GFAP and anti-GAPDH antibodies. Bars represent the average of GFAP/GAPDH from 4 mice per group ± SEM (*p<0.05). (B) Brain slices were analyzed by immunohistochemistry for reactive astrocytes using anti-GFAP antibodies. The total stained area of the thalamus region was quantified and normalised to that of PBS-injected wild-type mice. Representative GFAP positive cells are shown (photographed with ×40 objective). Bars represent the average of 3 mice per group ± SEM (*p<0.0001).

We have previously demonstrated that Eif2b^R132H/R132H^ mice exhibit delayed development of white matter in association with decreased abundance of brain astrocytes at post-natal day 21 (P21), the peak of myelin formation in mice. Moreover, mature mutant mice fail to recover from cuprizone-induced demyelination [24]. To further study the effect of the mutation in eIF2B5 on astrocyte function, we evaluated the extent of astrogliosis in response to LPS. Since VWM progressively deteriorates upon exposure to various stressors, including febrile illness, we used the pyrogenic agent LPS to induce fever and follow the increase in the brain levels of GFAP, which is considered a reliable marker for astrogliosis [25]. For this purpose, intraperitoneal injections of 5 mg/kg LPS or PBS were administered to 7 wild-type and 7 mutant mice, followed by western blot analysis of whole brains and immunostaining of brain slices at 1 week post-injection. Figure 1 demonstrates that although GFAP is induced in both control and mutant mice in response to the systemic inflammatory response, it reaches a significantly lower level in the brains of mutant compared to wild-type mice, indicating that astrogliosis is impaired due to the mutation in eIF2B5.

### eIF2B5-mutant Reactive Astrocytes are Dysmorphic and Overexpress GFAP-δ

**Figure 2:**
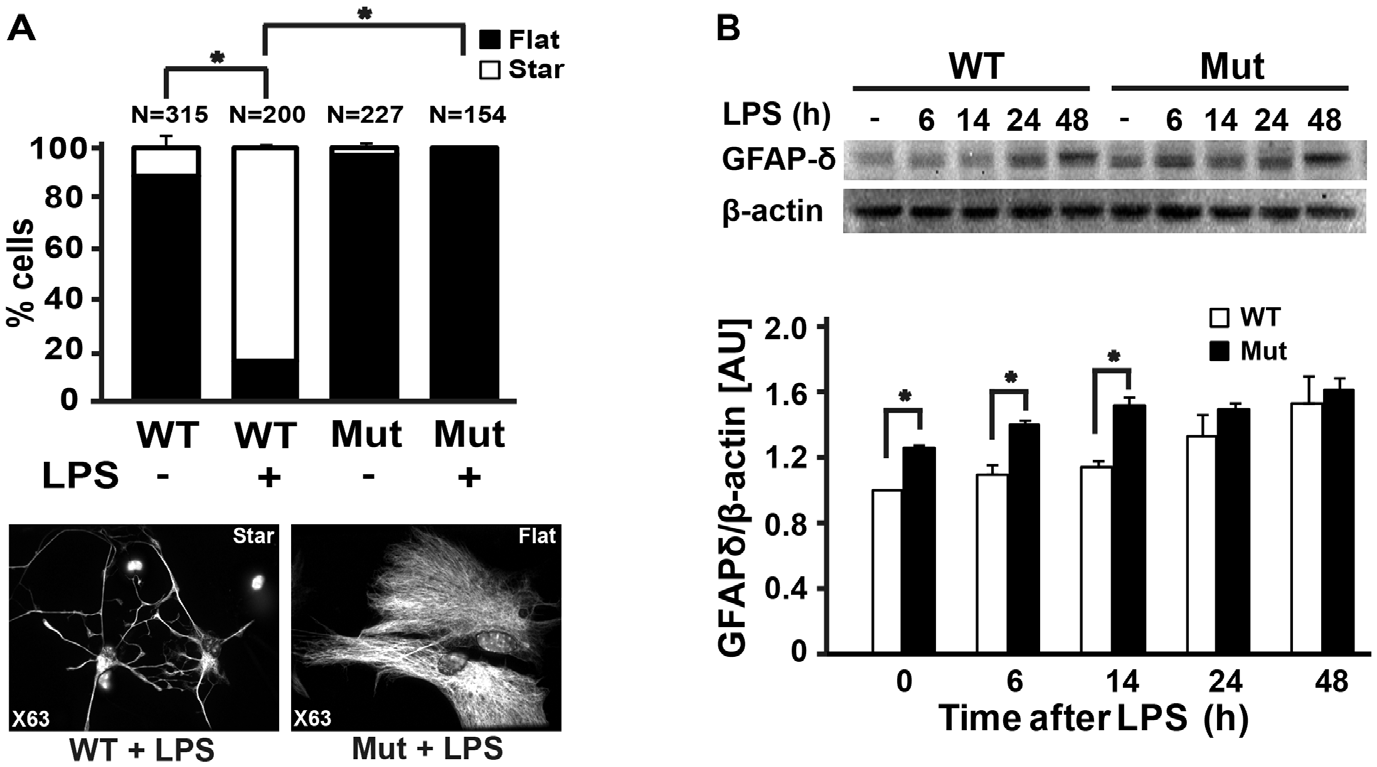
Eif2b5^R132H/R132H^ primary astrocytes exhibit abnormal morphology and overexpress GFAP-δ. (A) Primary astrocytes isolated from Eif2b5^R132H/R132H^ (Mut) and wild-type (WT) mice were incubated with 2 µg/ml LPS for 48 h followed by immunostaining for GFAP. Typical “flat” and “star” morphology is shown (photographed with ×63 objective). All cells within random fields (photographed with ×10 objective) were counted and assigned “star” or “flat” morphology (N = number of cells). Bars represent percent of “star” (white) or “flat” (black) morphology (*p<0.001). (B) WT and Mut primary astrocytes were treated with 2 µg/ml LPS for the indicated times, followed by immunoblot analysis of GFAP-δ and β-actin. GFAP-δ/β-actin levels were normalised to untreated WT cells. Bars represent the average of GFAP-δ/β-actin levels of 3 independent experiments ± SEM. (WT, white; Mut, black; *, p<0.005).

To investigate the effect of the mutation in eIF2B5 on astrocyte function, astrocytes were isolated and cultured from wild-type and mutant newborn mice. Primary astrocyte cultures were treated with LPS and visualised by fluorescent microscopy following GFAP staining. Upon LPS treatment, the morphology of wild-type astrocytes changes from “flat” to “star” shape (Figure 2A), as expected for normal activation [23]. In contrast, the morphology of mutant astrocytes is not similarly affected by LPS. Whereas 100% of LPS-treated wild-type astrocytes acquired the anticipated “star” shape, the majority of LPS-treated mutant astrocytes remained “flat” (Figure 2A). Since dysmorphic astrocytes from VWM patients express the δ-isoform of GFAP [13], we tested astrocytes for GFAP-δ level and found that it is significantly higher in mutant compared to wild-type cells. Moreover, due to the elevated baseline level of GFAP-δ in resting mutant astrocytes, LPS treatment had only a marginal effect on mutant cells compared to the significant increase in GFAP-δ elicited in wild-type astrocytes (Figure 2B).

### Induction of Brain IL-6 and IL-1β in Response to LPS is Deficient in Eif2b5^R132H/R132H^ Mice

**Figure 3:**
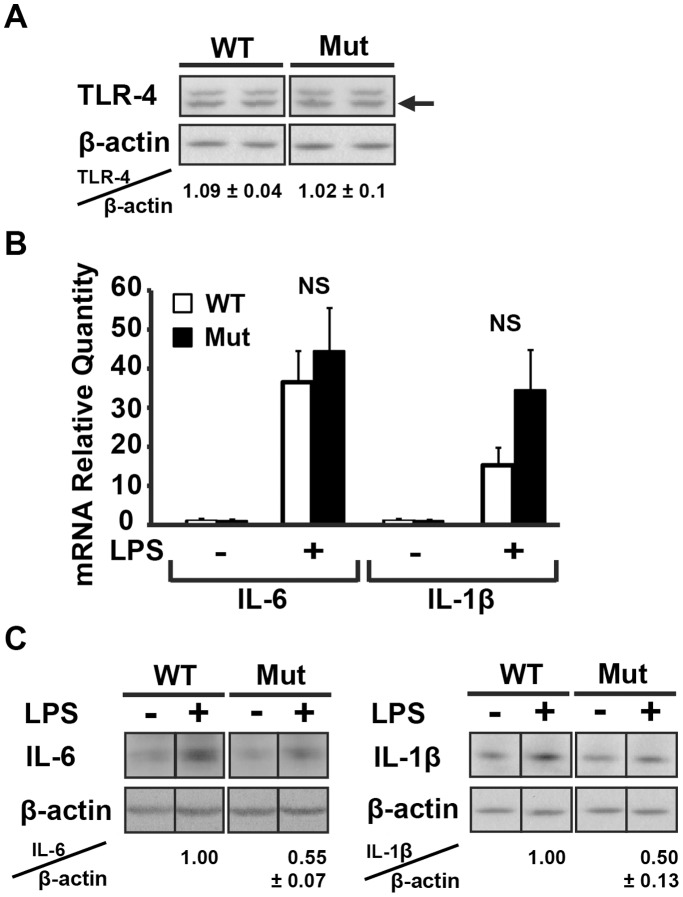
Induction of IL-6 and IL-1β in response to demand is impaired in Eif2b5^R132H/R132H^ mice brain. Intraperitoneal injections of 5 mg/kg LPS were administered to 4–6 four-weeks-old Eif2b5^R132H/R132H^ (Mut) and wild-type (WT) mice. At 6 h post-injection, mice were sacrificed and brains were removed. (A) Representative immunoblot analysis using anti-TLR4 and anti-β-actin antibodies. Data represent the average of TLR4/β-actin level of 3 mice per group ± SEM. (B) Total RNA was extracted and subjected to qRT-PCR analysis of IL-6 and IL-1β mRNA levels. Bars represent the average of 5 mice per group, normalised to untreated WT±SEM. No significant (NS) differences between Mut and WT were observed. (C) Representative immunoblot analyses using anti-IL-6, anti-IL-1β and anti-β-actin. Data represent the average of IL-6/β-actin and IL-1β/β-actin of 3-6 mice per group ±SEM relative to WT.

To further investigate the effect of the mutation in eIF2B5 on astrogliosis, we examined the levels of the pro-survival cytokines IL-6 and IL-1β, which are normally produced and secreted by activated glial cells to promote brain repair by stimulating migration of oligodendrocyte precursor cells (OPCs) and their differentiation to mature myelinating oligodendrocytes [26]. To test the effect of the mutation in eIF2B5 on the brain levels of IL-6 and IL-1β following insult, intraperitoneal injections of 5 mg/kg LPS were administered to 4-week-old wild-type and mutant mice, followed by qRT-PCR and immunoblot analyses of brain mRNA and protein at 6 hours post-injection, compared to non-injected controls. qRT-PCR analysis of IL-6 and IL-1β mRNA revealed that both transcripts are significantly elevated in the brain following LPS-induced systemic inflammatory response and reach similar levels in both wild-type and mutant brains (Figure 3B). This indicates that the initial steps of the toll like receptor 4 (TLR4) signalling pathway are not negatively affected in mutant mice, in line with the similar level of TLR4 itself observed in both groups (Figure 3A). However, although IL-6 and IL-1β mRNA levels were similarly induced in wild-type and mutant brains, immunoblot analyses revealed a significantly higher increase in IL-6 and IL-1β protein levels in wild-type compared to mutant brains following LPS treatment (Figure 3C).

### TLR4-dependent Inflammatory Response is Impaired in Mixed Glial Cell Cultures from Eif2b5^R132H/R132H^ Mice

**Figure 4:**
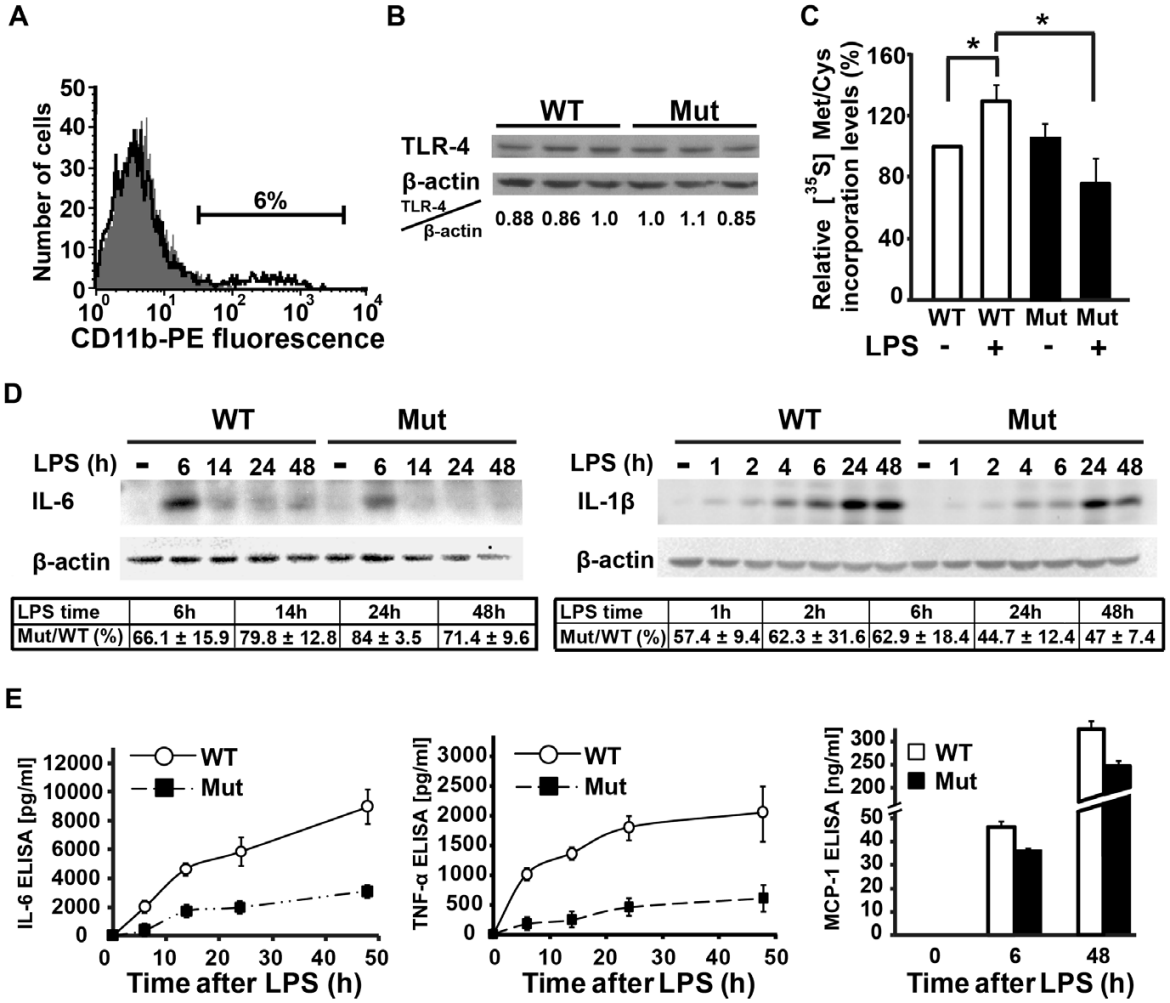
Activated primary astrocytes-microglia cultures from Eif2b5^R132H/R132H^ mice do not exhibit increased global translation and display impaired production of cytokines. Primary mixed glia cultures were isolated from Eif2b5^R132H/R132H^ (Mut) and wild-type (WT) mice. (A) Representative analysis of PE-labelled anti-CD11b staining followed by flow cytometry analysis. Both Mut and WT cultures contained 93–95% CD11b-negative (gray area) cells. (B) Immunoblot analysis of 3 independent experiments using anti-TLR4 and anti-β-actin antibodies. No statistically significant difference between WT and Mut cells was observed. (C) WT and Mut cells were treated with 2 µg/ml LPS for 48 h and metabolically labelled with [^35^S]L-methionine and [^35^S]L-cysteine for 30 min followed by determination of [^35^S]-Met/Cys incorporation following trichloroacetic acid precipitation. The incorporation level (cpm/µg protein) in untreated WT cells was set at 100%. Bars represent the means ± SEM of 3 independent experiments, *p<0.02. (D, E) WT and Mut cultures were incubated with 2 µg/ml LPS for the indicated times followed by cell harvest and media collection. A representative immunoblot analysis of intracellular IL-6 and IL-1β protein levels is shown and the average (of 3 independent experiments) of IL-6/β-actin and IL-1β/β-actin in Mut relative to WT is provided in (D); protein concentrations of IL-6, TNF-α and MCP-1 in the media was measured by ELISA and a representative of 3 independent experiments is shown in (E).

LPS induces transcription, translation and secretion of a wide range of proteins, including IL-6 and other cytokines and chemokines, via the TLR4 signalling pathway [27], [28]. To better understand which step of this cascade is adversely affected by the eIF2B5 mutation, we first verified the level of TLR4 and found that it is similar in primary glial cell cultures isolated from both wild-type and mutant mice brains (Figure 4B). In agreement with this observation, the mutation does not affect global protein synthesis rate in resting primary glial cell, as detected by a 30 minute pulse of [^35^S]-Methionine/Cysteine incorporation prior to LPS treatment (Figure 4C). However, upon LPS treatment, a significant increase in [^35^S]-Methionine/Cysteine incorporation was observed in the wild-type cells, whereas a similar increase was not observed in mutant cells (Figure 4C). To further test the ability of mutant cells to respond to TLR4 activation by producing specific proteins, cells were treated with LPS for 1 to 48 hours followed by immunoblot analyses of IL-6 and IL-1β, two cytokines known to be translated and secreted in response to LPS, which stimulate the production of neuroprotective mediators. Figure 4D demonstrates that after LPS treatment, wild-type cells have higher intracellular levels of both IL-6 and IL-1β compared to eIF2B5-mutant cells. We next used ELISA to determine the concentration of secreted IL-6, TNFα and MCP1 in the culture medium. TNFα and MCP1 are also known to be translated and secreted in response to LPS; TNFα promotes the inflammatory response and MCP1 is a chemoattractants that promotes migration of OPCs to demyelinated regions [29]. Figure 4E shows a marked difference in the concentration of all assayed proteins between mutant and wild-type cells, indicating that the mutation in eIF2B5 affects the function of activated primary glial cell and limits their ability to repair brain insults. While astrocytes are the major type of glial cell in the brain, microglia also serve an important function in brain repair. In response to insult, paracrine and autocrine networks stimulate a synergistic microglia-astrocyte crosstalk, which is critical for the outcome of repair [23]. Using anti-CD11b antibodies to detect microglia, we found that our primary cell cultures were highly enriched for astrocytes but still contained about 5–7% CD11b-positive microglia cells (Figure 4A). Therefore, we next sought to study the effect of the mutation in eIF2B5 on each of the cell types separately, under conditions of increased translational demand.

### Response to Increased Translational Demand is Deficient in Astrocytes from Eif2b5^R132H/R132H^ Mice

**Figure 5:**
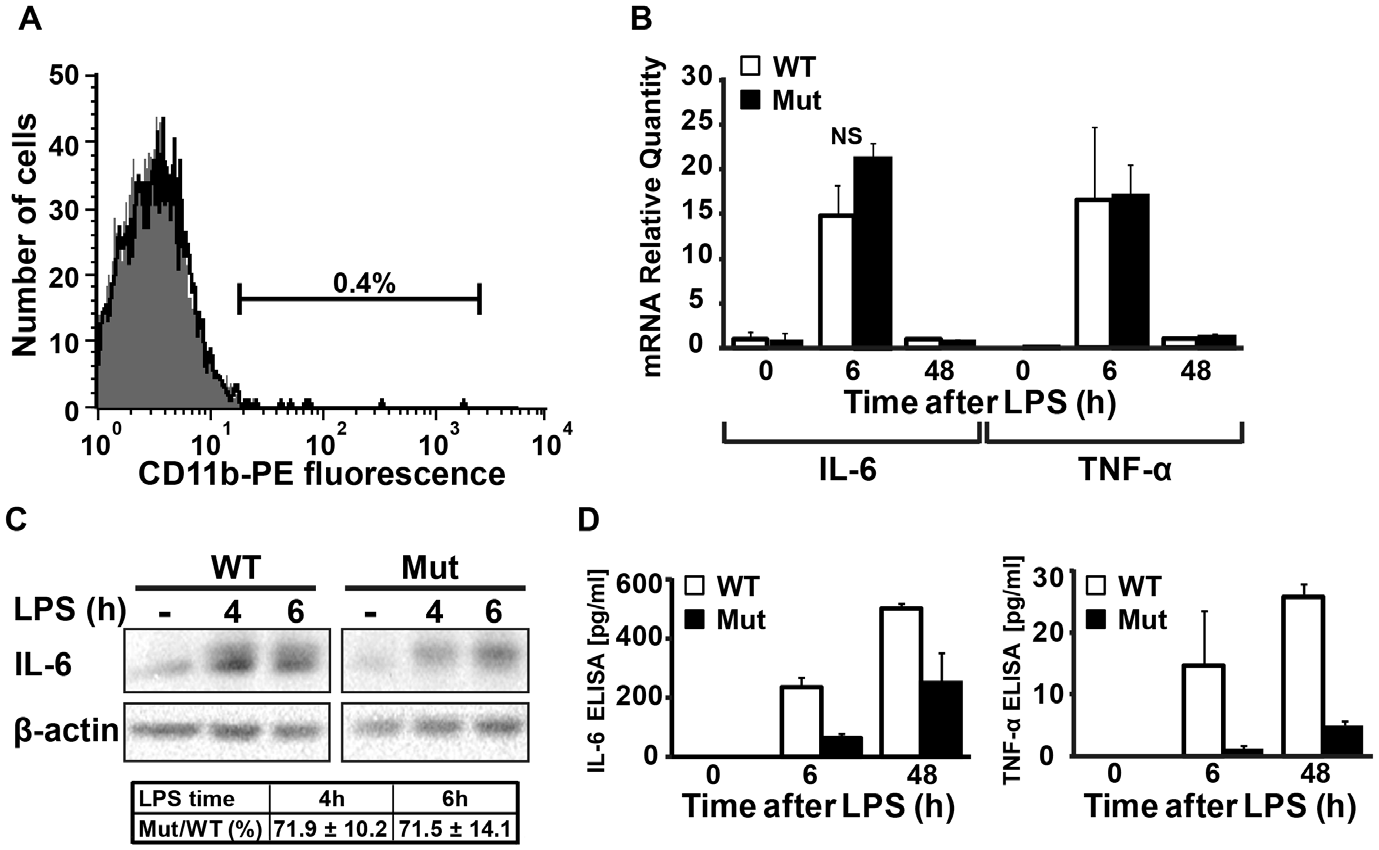
Activated Eif2b5^R132H/R132H^ primary astrocytes exhibit impaired production of cytokines. Primary astrocytes were isolated from Eif2b5^R132H/R132H^ (Mut) and wild-type (WT) mice. (A) Representative analysis of PE-labelled anti-CD11b staining followed by flow cytometry analysis. Both Mut and WT cultures were 99.4–99.7% CD11b-negative (gray area). (B,C,D) Both cultures were treated with 2 µg/ml LPS for the indicated times followed by media collection and cell harvest for RNA and protein extraction. qRT-PCR of IL-6 and TNF-α mRNA levels is shown in (B), bars represent the average of 3 independent assays, normalised to 48 h±SEM. No significant (NS) differences between Mut and WT were observed; intracellular IL-6 protein level was determined by immunoblot analysis with β-actin as loading control. A representative blot of 3 independent experiments is shown and the average of IL-6/β-actin in Mut relative to WT is provided in (C); protein concentrations of IL-6 and TNF-α in the media was measured by ELISA and a representative of 3 independent experiments is shown in (D).

Highly-enriched cultures of primary astrocytes were obtained by immunodepletion of CD11b-positive cells. This procedure yielded pure cultures with <0.5% microglia (Figure 5A). Primary wild-type and mutant astrocytes were then treated with LPS for various durations and harvested for extraction of intracellular RNA and protein, as well as secreted proteins, compared to untreated controls. qRT-PCR analysis of IL-6 and TNFα mRNA revealed that both transcripts are significantly elevated following LPS treatment and reach similar levels in both wild-type and mutant astrocytes (Figure 5B). This further supports the assumption that the initial steps of the TLR4 signalling pathway are not negatively affected in mutant astrocytes. While the levels of IL-6 mRNA were similarly elevated in wild-type and mutant astrocytes, immunoblot analysis revealed a higher increase in intracellular IL-6 protein levels in wild-type compared to mutant astrocytes following LPS treatment (Figure 5C), suggesting that translation of IL-6 mRNA under conditions of increased translational demand is impaired due to the mutation in eIF2B5. This is further supported by the lower concentration of secreted IL-6 and TNFα in the medium, as detected by ELISA. (Figure 5D).

### Response to Increased Translational Demand is Deficient in Microglia from Eif2b5^R132H/R132H^ Mice

**Figure 6:**
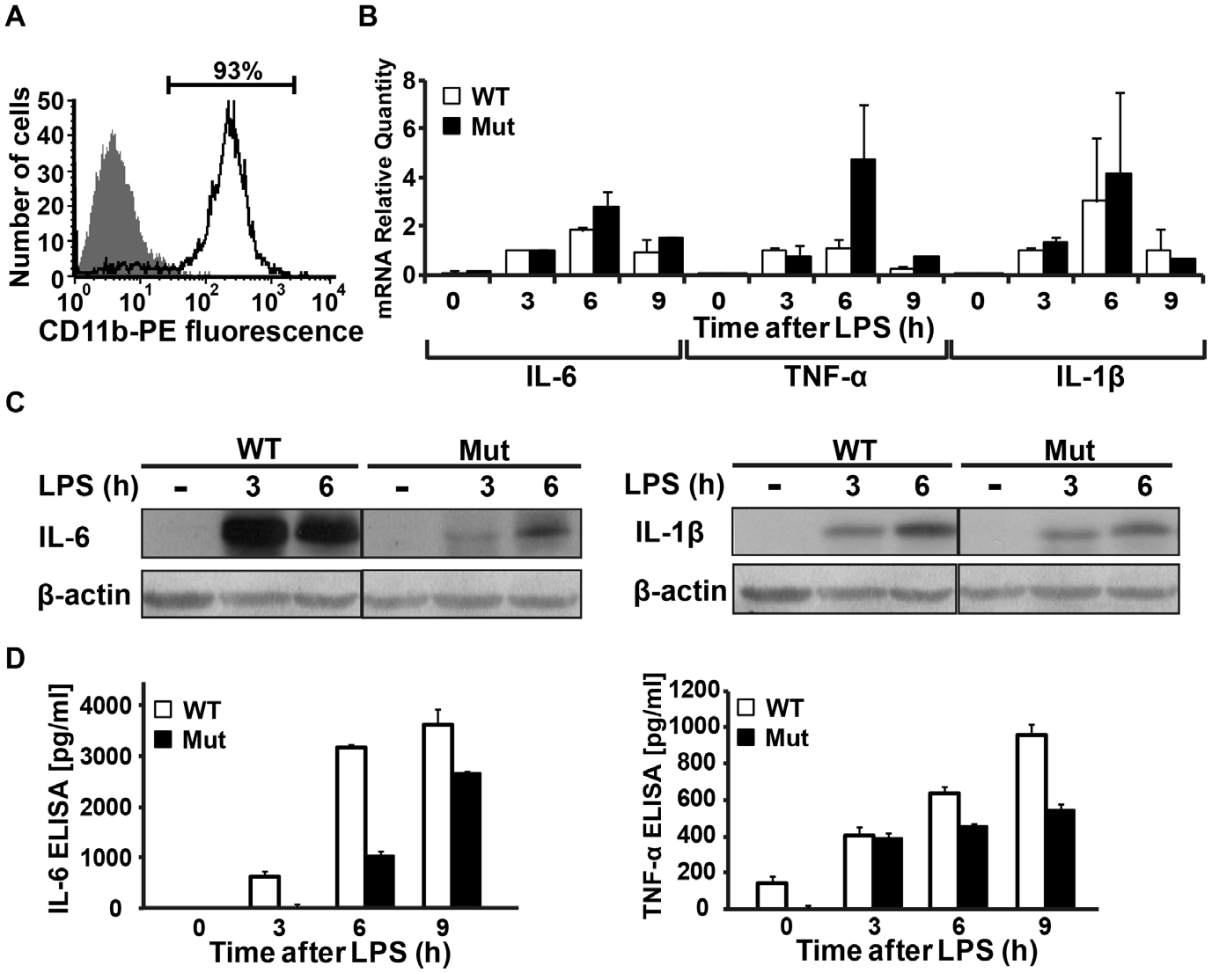
Activated Eif2b5^R132H/R132H^ primary microglial cells exhibit impaired production of cytokines. Primary microglial cells were isolated from Eif2b5^R132H/R132H^ (Mut) and wild-type (WT) mice. (A) Representative analysis of PE-labelled anti-CD11b staining followed by flow cytometry analysis. Both Mut and WT cultures were 92–95% CD11b-positive (white area). (B,C,D) Both cultures were treated with 0.1 µg/ml LPS for the indicated times followed by media collection and cell harvest for RNA and protein extraction. qRT-PCR of IL-6, TNF-α and IL-1β mRNA levels is shown in (B), bars represent the average of 3 independent assays, normalised to WT cells at 3 h of LPS treatment ± SEM. Intracellular IL-6 and IL-1β protein levels were determined by immunoblot analyses with β-actin as loading control, representative blots of 3 independent experiments are shown in (C); protein concentrations of IL-6 and TNF-α in the media was measured by ELISA and a representative of 3 independent experiments is shown in (D).

Next, microglial cells were isolated from newborn wild-type and mutant mice to obtain pure cultures of >92% CD11b-positive cells (Figure 6A). The primary wild-type and mutant microglia cultures were treated with LPS for various durations and harvested for extraction of intracellular RNA and protein, as well as secreted proteins, compared to untreated controls. qRT-PCR analysis of IL-6, TNFα and IL-1β mRNAs revealed that these transcripts are significantly elevated following LPS treatment and reach similar levels in both wild-type and mutant microglia (Figure 6B). However, while mRNA levels are similar, the intracellular levels of IL-6 and IL-1β proteins were significantly lower in mutant compared to wild-type microglia (Figure 6C), and so were the levels of secreted IL-6 and TNFα (Figure 6D). Again, similarly to the results observed in the astrocyte cultures, these data suggest that the translation of cytokines is impaired due to the mutation in eIF2B5.

## Discussion

### Astrogliosis in Response to LPS is Impaired in eIF2B5-mutated Mice

Post-mortem brains of human VWM patients revealed abnormal astrocyte morphology, anomalous composition of intermediate filament network and maturation defects [9]–[13]. Although these findings strongly suggest that eIF2B mutations are associated with impaired and atypical astrogliosis, they do not directly demonstrate a failure of eIF2B-mutant astrocytes to properly respond to activation signals. The Eif2b5^R132H/R132H^ knock-in mouse model for VWM provides a continuous source of fresh mutant glial cells with matching wild-type controls. Using this mouse model, we analyzed the effect of the mutation in eIF2B5 on the biological functions of both astrocytes and microglia in response to LPS, under controlled experimental conditions. The rate of protein synthesis in resting mutant astrocytes is normal based on [^35^S]-methionine/cysteine incorporation assays, despite a ∼20% reduction in GEF activity of mutant eIF2B in the brain [24], suggesting that the lowered GEF activity is sufficient to maintain the required translation rates under normal conditions. This assumption is further supported by the observation that TLR4 protein levels are normal in mutant brains and primary astrocytes (Figures 3 and 4) and that transcription of IL-6 and TNF-α is similarly induced in response to LPS treatment in both mutant and wild-type astrocytes (Figure 3 and 5), implying that the levels of downstream components of the TLR4 signalling pathway are normal. However, despite the normal induction of transcription, which is based on proteins that are synthesised under normal conditions, [^35^S]-Methionine/Cysteine incorporation assays revealed that mutant astrocytes fail to support efficient translation of mRNAs transcribed following LPS treatment (Figure 4). This is in agreement with the lower intracellular and secreted levels of IL-6 and TNF-α proteins following LPS treatment in mutant compared to wild-type astrocytes, despite similar levels of LPS-induced IL-6 and TNF-α mRNAs. Taken together, these observations suggest that the mild decrease in the enzymatic activity of eIF2B significantly hampers the ability of astrocytes to cope with acute demands for increased translation, resulting in impaired activation and deficient response to brain insults. The concept that the mutation in eIF2B5 is associated with poor astrocyte activation was also demonstrated at the whole-animal level, as GFAP levels were strongly induced in wild-type but not mutant brains following systemic injections of LPS (Figure 1). The significantly lower increase in the levels of GFAP, a key marker of astrogliosis [25], is clear evidence that the mutation in eIF2B5 impairs astrogliosis. Impaired astrocyte activation was also evident from their inability to change from “flat” to “start” morphology (Figure 2), which is in agreement with the abnormal morphology and blunt processes of astrocytes detected in brain sections from VWM patients [9], [10], [30], [31]. Interestingly, the dysmorphic mutant mice astrocytes express high baseline levels of the GFAP-δ isoform even prior to LPS treatment (Figure 2), reflecting a background pathological condition that can also be detected in astrocytes of VWM patients [13].

### eIF2B5 Mutation is Associated with Incomplete Astrocyte and Microglia Activation and Defective Inflammatory Response

In addition to the anomalous quantity and quality of intermediate filament networks and the related morphological outcome, the current study provides evidence that Eif2b5^R132H/R132H^ mice fail to synthesise increased amounts of IL-6 and IL-1β protein in the brain in response to systemic LPS injection despite similar levels of LPS-induced IL-6 and IL-1β mRNAs (Figure 3). This observation implies that a mild reduction in eIF2B activity can negatively affect the capacity of the brain to elicit repair functions. Restoring brain homeostasis in response to insults involves microglia-astrocyte crosstalk, in which repair-promoting cytokines and chemokines are produced and secreted by both glial cell types. The beneficial functions of microglia in successful transient activation of astrocytes towards brain repair and homeostasis are well characterized [32], [33]. The importance of microglia to the inflammatory response was also seen in our experiments since pure microglia cultures responded to LPS treatment more robustly than pure astrocyte cultures; mixed glia cultures secreted ∼10-fold more IL-6 compared to the pure astrocyte cultures after 6 hours of LPS treatment, demonstrating that the presence of 5–7% microglial cells in the mixed glia cultures gave the astrocytes a significant boost (Figures 4,5,6). The harmful effect of eIF2B mutation on the functions of both cell types is evident by the significantly lower levels of intracellular and secreted IL-6, TNF-α, IL-1β and MCP-1 in the eIF2B-mutant compared to wild-type cells at all time-points and across all experiments (Figures 4,5,6). The current study provides first evidence for the critical effect of eIF2B mutation on the function of microglia and strongly implicates this cell type in disease progression due to their inability to cope with the acute demand for synthesis and secretion of various factors. The poor activation of microglia is further amplified by the insufficient activation of astrocytes, which are the dominant players in cerebral neuroprotective innate immunity [34]. Cytokine-activated astrocytes produce energy substrates and trophic factors for neurons and oligodendrocytes, scavenge free radicals and excess glutamate, restore the blood-brain barrier and CNS ionic homeostasis, promote remyelination and neovascularization and stimulate neurogenesis from neural stem cells [35]. Astrocytes also produce MCP1 and other chemoattractants that promote migration of immune cells and OPCs from their resting positions in the brain parenchyma to the demyelinated plaque [29]. Cytokines e.g. TNFα, IL-1β, and IL-6 stimulate the production of neuroprotective mediators. IL-1β promotes oligodendrocyte generation and remyelination through the induction of CNTF and IGF-I, which promote the proliferation and differentiation of OPCs, regulate myelin synthesis in mature oligodendrocytes and support their survival. IL-6 also acts as an oligodendrocyte survival factor and supports neuroprotection in-vivo [26], [36]–[39]. Disturbance or loss of function during reactive astrogliosis has been reported to lead to pathological outcomes [22], [23], [26], [40]; here we demonstrate that proper regulation of the inflammatory response upon brain insult, which is crucial for brain repair, is compromised due to the mutation in eIF2B.

### Updated Thoughts on the Aetiology of eIF2B-leukodystrophy

Our work suggests that a specific mutation in eIF2B can have either a minor or major effect on cellular functions, depending on the status of the translational machinery in a specific cell type. We show that the R132H mutation in mouse eIF2B5 protein does not affect global translation rate in astrocytes under normal conditions, but prevents increased production of proteins upon LPS treatment. We also demonstrate that despite similar levels of TLR4 in eIF2B-mutant and wild-type cells, activation of the TLR4 signalling pathway fails to induce adequate synthesis of cytokines in eIF2B-mutant microglia and astrocytes. Based on these observations, we hypothesize that VWM disease may result from a loss-of-function of certain cell types under specific conditions due to inability to produce the necessary amounts of newly-synthesised proteins within a short timeframe. This is best exemplified in the mixed glia system, which relies on paracrine and autocrine feedback loops, where microglial- and astrocyte-derived factors activate and regulate each other in response to brain insult. The impaired activation of microglia and astrocytes in response to insult can explain their dysfunction in promoting remyelination and brain homeostasis and may shed light on the deterioration of clinical symptoms in VWM patients upon exposure to physiological stress. In line with this concept, another example of “acute” demand for increased translation, which may be intolerable in eIF2B-mutant cells, is the demand for massive synthesis of myelin components by oligodendrocytes within the short timeframe of normal myelination. The prolonged myelin production during early postnatal brain development of Eif2B5^R132H/R132H^ mice, their failure to overcome cuprizone-induced demyelination and their prolonged astrogliosis during the delayed repair period [24] support this concept. It has been shown recently that the severity of VWM symptoms does not correlate with the extent of reduction in eIF2B activity, as assayed in fibroblasts or transformed lymphocytes derived from patients with different mutations in eIF2B [41]. We suggest two possible explanations for this surprising discrepancy: (i) eIF2B GEF activity as well as eIF2 levels may not be identical in brain cells and fibroblasts or transformed lymphocytes; (ii) the disease severity may strongly depend on random exposure of each patient to brain insults during critical times of brain development or repair. According to the latter, following exposure to multiple stress events a mild mutation may be associated with severe clinical symptoms, whereas a more potent mutation may be associated with mild symptoms if the patient was fortunate to avoid brain damaging events during critical times. Human siblings who carry an identical eIF2B mutation but suffer divergent severity of clinical symptoms further substantiate the above ideas. The current study emphasizes the significance of exposure to physiological stress to the aetiology of eIF2B-related leucodystrophy.

## Materials and Methods

### Mice Maintenance

Mice were housed in an animal facility with a 14/10 h light/dark cycle in groups of four to seven animals in each filtered-top cage supplemented with autoclaved wood chips in laminar flow hoods. Animals were fed autoclavable rodent pellet (Koffolk 19–510, Koffolk Ltd, Petach Tikva, Israel) and sterile water ad libitum throughout the experiments. All experimental procedures involving mice were approved by the Tel Aviv University Animal Care Committee according to national guidelines (permit #L-09-35).

### Lipopolysaccharide (LPS) Injection Followed by Immunohistochemistry or Immunoblot Analysis

5 mg/Kg of LPS (Sigma L4391) were injected into the peritoneum of mice at P14 and again at P15. Injection of phosphate-buffered saline (PBS) was used as a control. At P21 mice were anesthetized with 10% Ketamine/6.6% Xylazine in (PBS) and perfused transcardially with PBS followed by fixation with PFA fixation solution (4% paraformaldehyde in 0.1 M phosphate buffer, pH 7.4). Brains were removed, incubated overnight in PFA fixation solution followed by incubation in 30% sucrose for 48h. Frozen coronal sections (30 µm) were cut using a sliding microtome and collected serially. The free-floating sections were washed with 100 mM PBS (pH 7.4) prior to the addition of anti GFAP antibodies (Dako) diluted 1∶2000 in PBST (0.1% Triton X-100 in PBS) containing 2% goat serum. The sections were incubated 30 min at 37°C and then overnight at 4°C, rinsed with PBST, and incubated with diluted biotinylated anti-rabbit IgG (Vectastain kit #PK6100, Vector Labs) for 60 min at room temperature. After several rinses with PBST, sections were further incubated for 30 min in avidin-biotin-horseradish peroxidase complex (ABC Elite; Vector Laboratories) in PBST, rinsed with PBST, and soaked in diaminobenzidine-chromagen solution (Vector Labs). The reaction was monitored visually and stopped by rinses with PBS. To minimize variability, sections from all animals were stained simultaneously. Following dehydration and mounting using Histomount (Invitrogen), the peroxidase-immunostained sections were photographed using a 40X objectives and a Nikon DS-FI1 camera (Nikon Instech, Tokyo, Japan), followed by measurements of total stained area using Image-Pro Plus software (version 5.1 Media Cybernetics, Silver Spring, MD). Other than moderate adjustments of contrast and brightness, the images were not manipulated. Statistical comparison was performed using student’s t-test (one tailed distribution with equal variance). For immunoblot analysis of IL-6 and IL-1β, 4-weeks-old male mice were used for a single injection and were sacrificed 6 hours later as described above. Immediately after the perfusion, brains were removed from the skull and stored in −80°C until proteins were extracted from each individual brain by brief sonication in lysis buffer containing 20 mM Hepes (pH 7.7), 100 mM KCl, 10% glycerol, 2% Triton X-100, 20 mM β-glycerol phosphate, EDTA-free protease inhibitor cocktail (Complete™; Roche), and 1 mM dithiothreitol (DTT).

### Immunoblot Analysis

Cells were lysed in lysis buffer (20 mM HEPES (pH 7.5), 1% Triton, 100 mm KCl, 10% Glycerol, 50 mm NaF, 10 mm β-glycerol phosphate, 0.5 M DTT, 0.1 M Vanadate, EDTA-free protease inhibitor mixture (Complete, Roche), and 0.1 µm microcystin). Western blot analyses were performed following 12% SDS-PAGE according to standard procedures. Rabbit polyclonal anti IL-6 and IL-1β and goat polyclonal anti TNF-α were from Peprotech. Rabbit polyclonal anti GFAP-δ and anti TLR-4 were from Abcam, mouse anti GFAP from BD bioscience and mouse monoclonal anti β-actin was from Sigma. HRP-conjugated goat anti-mouse and anti-rabbit secondary antibodies were from Jackson Immuno Research Laboratories, Inc. Detection was by enhanced chemiluminescence (GE Healthcare). Densitometry of protein bands was performed using ImageJ software.

### Preparation of Primary Astrocytes Cultures

Brains from 6–9 P1-3 neonatal mice were collected in L-15 Leibovitz medium (Biological industries, Israel). Following removal of the meninges, the cortexes were dissected, washed with cold HBSS (Biological Industries, Israel), and digested for 5 min at room temperature in15 ml of 0.25% trypsin (Sigma # T9201) and 100 µg/ml DNase I type IV (Sigma # D5025) in DMEM (Biological Industries, Israel) without serum. Digestion was stopped by addition of 15 ml cold DMEM containing 10% heat inactivated Fetal Calf Serum (HI-FCS), 100 U/ml penicillin and 100 µg/ml streptomycin, followed by filtration through 100 µm nylon membrane (BD Falcon). After centrifugation (1100 rpm, 10 min, 4°C), the cells were resuspended in warm growth medium containing DMEM-10% HI-FCS supplemented with 2 mM L-Glutamine, 100 U/ml Penicillin, 100µg/ml Streptomycin, Non-Essential Amino Acids and 110 µg/ml Na-Pyruvate (all from Biological Industries, Israel), and seeded on T75 flasks pre-coated with 10 µg/ml of Poly-L-lysine (Sigma # P2636) at a density of ∼4×10^6^ live cells per flask. The growth medium was replaced at day 2 and day 7 after plating. At day 13 the flasks were shaken on an orbital shaker at 150 rpm for 18 hours at 37°C in the CO_2_ incubator. The remaining astrocytes and microglia were trypsinized with 0.25% trypsin (Biological Industries #03-052-1) and further plated on plates at a density of 1.8×10^4^cells/cm^2^. After approximately 10 days in culture, a sample was analyzed by flow-cytometry using anti CD11b antibodies. For this analysis, cells were gently removed from the plates by trypsin digestion, wash with flow cytometry buffer (1% BSA and 0.05% sodium azide in PBS), centrifuged at 1100 rpm for 5 min at 4°C, resuspended in flow cytometry buffer supplemented with PE-conjugated anti-CD11b mAb (BioLegend, USA) and incubated for 45 min at 4°C, followed by FACS analysis. The percent of CD11b-positive microglial cells was about 5–7%. To achieve more pure astrocytes cultures, microglial cells were removed by CD-11b immuno-depletion with MACS magnetics beads after the shaking stage according to MACS separation kit protocol (MiltenyiBiotec, #130-093-634). The final percent of microglia following this procedure was less than 0.5%.

### Preparation of Primary Microglia Cultures

Primary microglia were prepared as previously described [42], with minor modifications. Cerebral cortexes from 9–13 P1-3 neonatal mice were dissected in ice-cold HBSS (Biological Industries #02-016-1A), washed with cold HBSS, and digested with 5 ml 0.25% trypsin (GIBCO #25200-072) for 10 min at room temperature. Trypsinization was stopped by addition of an equal volume of growth medium (DMEM supplemented with 10% HI-FCS, 100 U/ml penicillin, and 100 µg/ml streptomycin) to which DNase I type IV (Sigma #D5025) was added to final concentration of 100 µg/ml. Following 1–2 min at room temperature (until DNA viscosity disappear), the cells were dispersed into a single-cell level by repeated pipetting, then pelleted (1100 rpm, 10 min, 4°C), resuspended in growth medium, and filtrated through 100 µm nylon membrane. The cells were seeded at a density of 1.6×10^6^ cells per 60 mm plate pre-coated with 10 µg/ml Poly-L-lysine (Sigma #P2636). The growth medium was replaced every 4–5 days. The mixed glia cultures reached confluency after 7–10 days and were further incubated without splitting. Between day 20–23 after plating, microglial cells were isolated by a mild trypsinization procedure as previously described [43]. Briefly, treatment of the confluent mixed glial cultures with 0.06% trypsin (GIBCO #25200-072) resulted in detachment of an intact layer of cells containing almost all the astrocytes and leaving behind a highly enriched population of microglia (>93%, as determined by flow cytometry analysis using CD11b antibodies, see above). The attached microglial cells were recovered for 24 hours and then were subjected to the different treatments.

### ELISA

ELISA development kits for murine IL-6, TNF-α and MCP-1 (#900-K50, #900-K54, and #900-K126, respectively; PeproTech Inc.) were used with recombinant proteins for standard curves.

### Quantitative Real-time PCR

Total RNA was extracted from mice brain and from primary cultures using TRIZOL reagent (Invitrogen). The final RNA concentration was measured using a NanoDrop ND-1000 spectrophotometer. First-strand cDNA was synthesised using Verso cDNA Kit (Thermo Scientific). Syber-based reactions were carried out for 40 cycles in StepOne Real-time PCR apparatus (Applied Biosystems) using Fast Syber® Green Master Mix (Applied Biosystems) with the following oligonucleotide primers: for IL-6 (mIL-6 Fwd5′-ATG GAT GCT ACC AAA CTG GAT-3′ and mIL-6 Rev 5′-TGA AGG ACT CTG GCT TTG TCT-3′); TNF-α (mTNF-α Fwd5′-CCC AGA CCC TCA CAC TCA GAT C-3′ and mTNF-α Rev 5′- CCT CCA CTT GGT GGT TTG CT -3′); IL-1β (mIL-1β Fwd5′-CTC CAT GAG CTT TGT ACA AGG-3′ and mIL-1β Rev 5′- TGC TGA TGT ACC AGT TGG GG-3′); and GAPDH which was used an internal control (mGAPDH-Fwd 5′TGGCAAAGTGGAGATTGTTGCC3′ and mGAPDH-rev 5′AAGATGGTGATGGGCTTCCCG3′).

### Protein Synthesis Rate Assay

Cells in three 70–80% confluent wells of a 6 well-plate were used for each measurement point. The cells were washed twice with PBS and incubated for 45 min with 0.75 ml/well of cysteine/methionine-free DMEM (Sigma# D0422) supplemented with 10% dialyzed FCS for starvation. Then, [^35^S]L-Met/[^35^S]L-Cys mix (NEN, #NEG072) was added to a final concentration of 20 µCi/ml for further 30 min incubation, followed by three washes with cold PBS. Cells were scraped off the plates by rubber scrapper with cold PBS, transferred to an eppendorf tube and centrifuged at 2800 rpm for 5 minutes at 4°C. Cell pellets were lysed with 70 µl PBS containing 1% triton and protease inhibitor cocktail (Complete™, Roche) for 30 min on ice and then centrifuged for 30 min at 14000 rpm at 4°C. Cytoplasmic protein concentration in the supernatant was analysed by Bradford assay. 15µl of each sample were applied onto 3 MM filter papers (Whatman) and washed three times for 1 min in boiling 5% (W/V) trichloroacetic acid containing traces of cold L-methionine and L-cysteine. The filters were then rinsed once in ethanol, dried and counted in a scintillation counter (Beckman).

### Immunofluorescence

3×10^5^ astrocytes per each well of a 24-well plate were seeded on coverslips pre-coated with 10 µg/ml Poly-L-Lysine (Sigma # P2636) and incubated overnight. On the next day, cells were treated with 2 µg/ml LPS for 48 hours, rinsed with PBS and fixed by 4% paraformaldehyde in PBS for 10 min at room temperature. Cells were then permeabilised with PBS containing 0.1% Triton for additional 10 min followed by blocking in PBS containing 4% BSA for 20 min at room temperature. Incubation with anti GFAP antibody (DAKO# Z0334) diluted 1∶200 in PBS-4% BSA was performed for 60 min at room temperature, followed by three washes with PBS and further incubation for 60 min with anti-rabbit biotin (Amersham Biosciences #RPN1004) diluted 1∶200 in PBS-4% BSA, and Hoechst (Sigma). Cover slips were then washed three times and incubated for 45 min with StrepAvidin–Texas red (Amersham Biosciences #RPN1233) or StrepAvidin–FITC (Amersham Biosciences #RPN1232) both diluted 1∶100 in PBS-4% BSA. Cells were imaged using a spinning disk confocal microscope (Yokogawa CSU-22 Confocal Head; Axiovert 200 M, Zeiss, CoolSNAP HQ-CCD camera, 10x, 63x lens all under the command of Slidebook^Tm^) which was also employed for data analysis. Images of 10x magnification were used for manual counting of star and flat shaped astrocytes.
